# Antiproliferative Effects of Cucurbitacin B in Breast Cancer Cells: Down-Regulation of the c-Myc/hTERT/Telomerase Pathway and Obstruction of the Cell Cycle

**DOI:** 10.3390/ijms11125323

**Published:** 2010-12-22

**Authors:** Suwit Duangmano, Sumana Dakeng, Weena Jiratchariyakul, Apichart Suksamrarn, Duncan R. Smith, Pimpicha Patmasiriwat

**Affiliations:** 1 Faculty of Medical Technology, Mahidol University Main Campus, 999 Puttamonthon 4 street, Salaya, Nakornpathom 73170, Thailand; E-Mails: sduangmano@hotmail.com (S.Du.); sdakeng@yahoo.com (S.Da.); 2 Faculty of Pharmacy, Mahidol University, Bangkok, Thailand; E-Mail: pywju@mahidol.ac.th; 3 Faculty of Science, Ramkhamhaeng University, Bangkok, Thailand; E-Mail: S_apichart@ru.ac.th; 4 Institute of Molecular Biosciences, Mahidol University, Bangkok, Thailand; E-Mail: duncan_r_smith@hotmail.com

**Keywords:** cucurbitacin B, telomerase, hTERT, c-Myc, breast cancer, estrogen receptor

## Abstract

Naturally occurring cucurbitacins have been shown to have anticancer, antimicrobial and anti-inflammatory activities. In this study, we determined the effects of cucurbitacin B extracted from the Thai herb *Trichosanthes cucumerina L.* on telomerase regulation in three human breast cancer cell lines (T47D, SKBR-3, and MCF-7) and a mammary epithelium cell line (HBL-100). Cell viability after treatment with cucurbitacin B, which is an active ingredient of this herb, was assessed. Telomeric Repeat Amplification Protocol (TRAP) assays and RT-PCR (qualitative and realtime) were performed to investigate activity of telomerase as well as expression of human telomerase reverse transcriptase (hTERT) and c-Myc. The c-Myc protein level was also determined in SKBR-3 and HBL-100 cells. Our results show that the cucurbitacin B inhibits growth and telomerase activity in the three breast cancer cell lines and exerts an obvious inhibitory effect in the estrogen receptor (ER)-negative breast cancer SKBR-3 cells. The expression of hTERT and c-Myc were also inhibited by cucurbitacin B, In addition, a clear reduction of c-Myc protein was observed after treatment in SKBR-3 cells even with a concentration of cucurbitacin B that was ten-times lower compared to the concentration used for HBL-100. Our findings imply that cucurbitacin B exerts an anticancer effect by inhibiting telomerase via down regulating both the hTERT and c-Myc expression in breast cancer cells.

## 1. Introduction

The rising incidence of breast cancer makes this disease a major public health problem and breast cancer is the most common female cancer worldwide [1,2]. Telomerase, the enzyme responsible for maintaining telomeres, is highly expressed in over 90% of breast cancer tumors, but the expression is low in normal breast tissue [3,4]. Telomeric TTAGGG tandem repeats at the ends of chromosomes protect the chromosomes from end-to-end fusion, rearrangement and translocation [5,6]. In normal somatic cells with low telomerase levels, DNA polymerase cannot completely replicate the 5′ end of the lagging strand, leading to telomere shortening with each cell division. This telomere shortening triggers the cell to undergo replicative senescence, cell cycle arrest and DNA damage signaling [7,8]. Telomerase consists of two core subunits required for its activity: the human telomerase RNA template (hTERC) and the catalytic subunit of human telomerase reverse transcriptase (hTERT) [9,10]. Telomerase plays a role in cell proliferation as a protective mechanism against end-replication problems by adding TTAGGG repeats to the telomeres [11]. Telomerase is highly active only in germ cells and in tissue stem cells. Increased expression of telomerase is strongly associated with neoplastic growth and the expression of telomerase in cancer cells may be a necessary step for tumor progression. Therefore, the development of agents having activity against telomerase may be a productive approach to develop novel breast cancer therapies [12–14]. Chemotherapeutic drugs are quite toxic for patients and often result in drug resistance after a certain period. The use of alternative therapies is widespread in all regions of the developing world, and is rapidly growing in industrialized countries [15]. Natural cucurbitans are highly oxygenated, tetracyclic triterpenes containing the cucurbitane nucleus skeleton and are predominantly found in plants of the family cucurbitaceae, members of which have long been used in oriental medicines. Cucurbitacin B and dihydrocucurbitacin B extracted from the Thai herb *Trichosanthes cucumerina L*. have been shown to have anticancer, antimicrobial and anti-inflammatory activities [16,17] and the structures of these purified forms have been elucidated using NMR spectroscopy [18]. A previous report has shown that cucurbitacin B and dihydrocucurbitacin B can inhibit the growth of breast cancer cell lines [19], but the molecular mechanism of this growth inhibition remains unclear. Therefore, in the report herein, we addressed the effects of cucurbitacin B on telomerase and associated genes in human breast cancer cell lines. Our findings strongly suggest the potential anti-breast cancer effect of cucurbitacin B.

## 2. Results and Discussion

### 2.1. Effect of Cucurbitacin B on the Viability of Human Breast Cancer Cells

**Figure 1.**
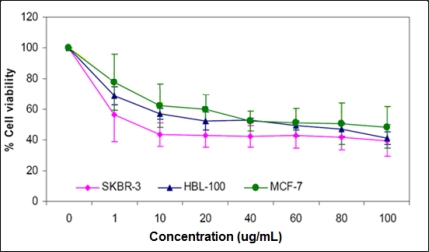
Effect of cucurbitacin B on the growth of human breast cancer cells. SKBR-3, MCF-7 and HBL-100 were treated with cucurbitacin B at final concentrations of 1, 10, 20, 40, 60, 80 and 100 μg/mL. Cell viability was determined by the MTT assay. The percentage of viable cells was calculated by defining the absorption of cells without cucurbitacin B treatment as 100%. Results are the average from three independent experiments.

To investigate the effects of cucurbitacin B on human breast cancer cells, IC_50_ values for three extracts of *T. cucumerina L* were determined on breast cancer cell lines. The extracts used included a 100% pure cucurbitacin B and a purified cucurbitacin B compound containing cucurbitacin B (major) and dihydrocucurbitacin B in 8:1 molar ratio. All were prepared as described elsewhere [19]. IC_50_ values of these purified extracts were also compared with the spray-dried crude extract. SKBR-3, MCF-7, T47D and HBL-100 cells were incubated with concentrations of the three testing extracts, ranging between 1 and 100 μg/mL and assessed for cell viability using the MTT assay at 48 h post incubation. All three extracts showed a significant growth inhibitory effect against SKBR-3 and HBL-100 cells, but less effect against MCF-7 and T-47D cells (Table 1 and Figure 1), suggesting that estrogen receptor (ER) negative cells are more sensitive to the action of these extracts.

Given that very similar, albeit only slightly lower inhibitory effects were observed with the cucurbitacin B compound (containing both cucurbitacin B and dihydrocucurbitacin B) as compared with purified cucurbitacin B, further experiments were performed to investigate the mechanism of action with the cucurbitacin B compound.

**Figure 2.**
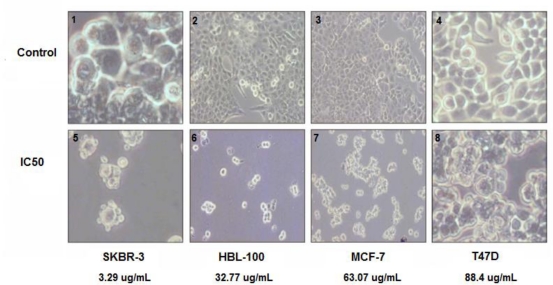
Morphological changes in breast cancer cells induced by cucurbitacin B. Upper (1, 2, 3, and 4): Phase-contrast photomicrographs of untreated SKBR-3, HBL-100, MCF-7 and T47D cells. Lower (5, 6, 7, and 8): SKBR3, HBL-100, MCF-7 and T47D cells treated with cucurbitacin B at IC_50_ for 48 h.

To confirm the loss of cell viability observed in cells incubated with the cucurbitacin B, all four cell lines were incubated with the extract at the calculated IC_50_ values as indicated in Table 1 for 48 h, in parallel with the control (untreated) cells. Examination of cultures by phase contrast microscopy showed morphological changes, including cell shrinkage and membrane blebbing, after treatment with the extract. This observation is consistent with the induction of apoptosis in the treated cells (Figure 2) and supports the loss of cell viability as documented in the MTT assay.

### 2.2. Effect of Cucurbitacin B on Telomerase Activity

**Figure 3.**
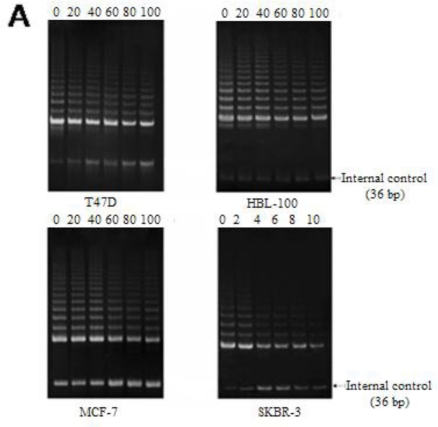

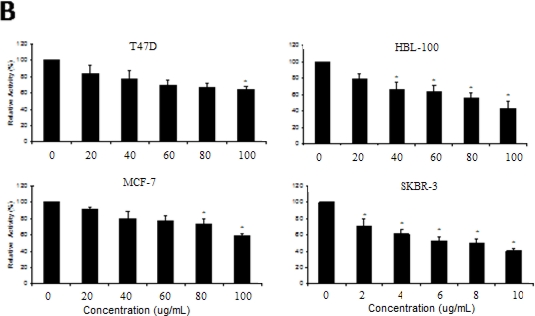
Effect of cucurbitacin B on telomerase activity in breast cancer cells. (**A**) SKBR-3 cells were treated with cucurbitacin B at final concentrations of 0, 2, 4, 6, 8 and 10 μg/mL while T47D, MCF-7 and HBL-100 cells were treated with cucurbitacin B at final concentrations of 0, 20, 40, 60, 80 and 100 μg/mL. Telomerase activity was examined by the TRAP assay and the decrease in telomerase activity was interpreted by means of reduced intensities of bands (stepwise 6 bp (TTAGGG) repetitive ladder bands). (**B**) Relative telomerase activities of the cells after cucurbitacin B treatment were determined by the TRAPEZE-ELISA assay, and the relative telomerase levels are expressed as % relative activity. * *P* < 0.05 *vs*. control (untreated) group.

The effect of cucurbitacin B on telomerase activity was investigated. The human breast cancer cell lines were treated with cucurbitacin B at varying concentrations for 48 h. According to the markedly greater sensitivity of SKBR-3 cells to cucurbitacin B compound, the SKBR-3 cells were, therefore, incubated at 10-fold lower concentrations than the other three cell lines. After incubation, telomerase activity was determined by the TRAP assay. As shown in Figure 3A, the band intensities of the 6 bp (TTAGGG) repeat length were reduced after incubation with cucurbitacin B in all cell lines tested, and the results are consistent with a dose dependent reduction of telomerase activity in the four cell lines. To confirm this, relative telomerase activity levels were determined using the TRAPEZE-ELISA detection assay system, and a dose dependent reduction in telomerase levels was observed in all four cell lines (Figure 3B). Both the assays for telomerase activity showed a greater effect of the cucurbitacin B in the estrogen receptor negative cell lines (SKBR-3 and HBL-100) as compared to the estrogen receptor positive cell lines (MCF-7 and T47D), consistent with the results from the cell viability assays. Moreover, telomerase activity was obviously reduced in ER negative breast cancer SKBR-3 cells, even though concentrations of cucurbitacin B used for this cell type were 10-times less than the non-malignant mammary epithelium HBL-100.

### 2.3. Effect of Cucurbitacin B on hTERT and c-Myc Gene Expression

**Figure 4.**
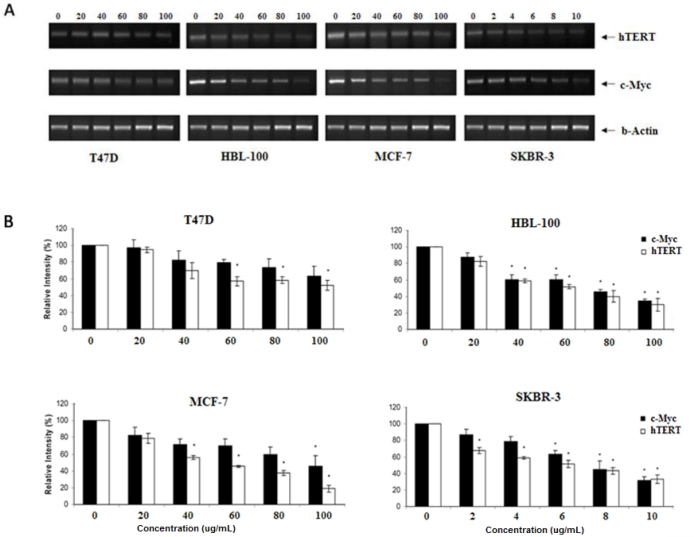
Expression.of *hTERT* and *c-My*c by RT-PCR. T47D, HBL-100, MCF-7 and SKBR-3 cells were incubated for 48 h with the specified concentrations of cucurbitacin B and RNA was extracted for RT-PCR. Levels of hTERT and c-Myc expression were analyzed. Bands representing hTERT and c-Myc are shown (**A**) and the quantitative intensities of each band after normalization with β-actin (**B**). * *P* < 0.05 *vs*. control (untreated) group.

To further determine the mechanisms of telomerase inhibition by cucurbitacin B compound, the expression of two genes (hTERT and c-Myc) known to affect telomerase activity were analyzed by semi-quantitative and quantitative realtime RT-PCR. For semi-quantitative RT-PCR, the cell lines were incubated with cucurbitacin B at concentrations of 0, 20, 40, 60 and 80 μg/mL (T47D, MCF-7 and HBL-100) or 0, 2, 4, 6, 8 and 10 μg/mL (SKBR-3) for 48 h and each experiment was undertaken in triplicate. Results, after normalization against β-actin, are consistent with a dose dependent reduction of both hTERT and c-Myc transcripts in all four cell lines. Again, SKBR-3 shows the greatest reduction of hTERT and c-Myc expression after cucurbitacin B treatment (Figure 4).

**Figure 5.**
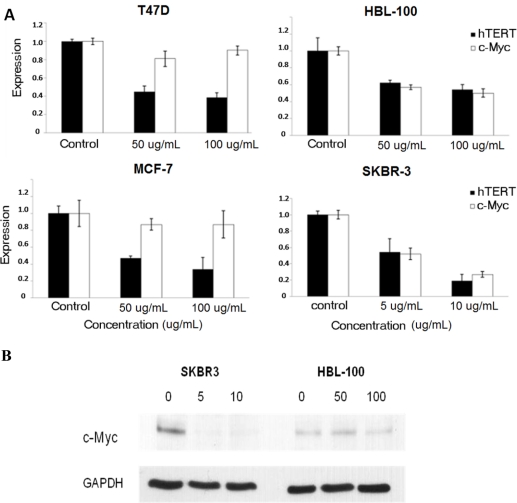
Expression of *hTERT* and *c-My*c. (**A**) T47D, HBL-100, MCF-7 and SKBR-3 cells were incubated with the specified concentrations of cucurbitacin B for 48 h and the quantitative expression levels of *hTERT* and *c-My*c were analyzed by realtime RT-PCR as described in the text. Results shown are the average of three independent experiments. (**B**) c-Myc protein expression. SKBR-3 and HBL-100 were treated with cucurbitacin B for 24 h. The cell lysates were separated by SDS-PAGE and analyzed by Western blot.

Realtime RT-PCR was undertaken to quantitate levels of the transcripts. For each cell line, only two concentrations of cucurbitacin B were tested in parallel with untreated cells: 50 and 100 μg/mL (for T47D, MCF-7 and HBL-100) or 5 and 10 μg/mL (for SKBR-3), and incubations were performed for 48 h as mentioned above. Results from the e RT-PCR (Figure 5(A)) confirm that cucurbitacin B from *T. cucumerina L*. inhibits gene expression of *hTERT* and *c-Myc*. Expression of *hTERT* and *c-Myc* in SKBR-3 cells was again reduced the most, even at the lower cucurbitacin B concentration of 5 to 10 μg/mL. The expression of c-Myc was mildly reduced in the estrogen positive cell lines (T47D and MCF-7), in contrast to the hTERT expression, in which the obvious reduction was also seen in these cells. Reduced c-Myc protein level in SKBR-3 cells is shown in Figure 5(B).

### 2.4. Effect of Cucurbitacin B on Cell Cycle Progression

**Figure 6.**
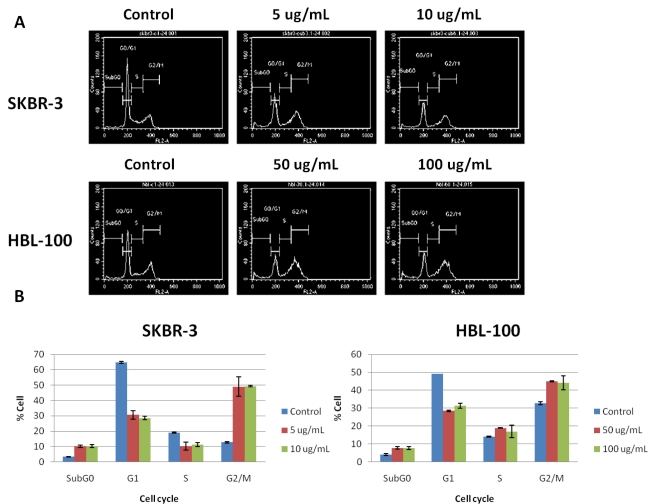
Effect of cucurbitacin B on the cell cycle in human breast cancer cells. SKBR3 and HBL-100 cells were incubated with cucurbitacin B for 24 h. The distribution of cell cycle stages was detected by propidium iodide (PI) staining. Results shown are the average of three independent experiments (**A**). Percent cells in each cell cycle phase represented as bar-graphs (**B**). Blockage at G2/M and apoptotic induction were observed in SKBR-3 after incubated with low concentration (5, 10 μg/mL) of cucurbitacin B.

Effect of cucurbitacin B on cell cycle progression in ER negative SKBR3 and HBL-100 were analyzed according to the principle of the cells’ DNA content in each phase of cell cycle. The cells were treated with cucurbitacin B for 24 h and DNA content was analyzed via flow cytometry. The experiment was performed in triplicate. Results shows that the treated cells were arrested at the G2/M phase of cell cycle with decreased cell population in G1 and S (Figure 6), suggesting that cucurbitacin B inhibits growth via blocking the cell division cycle at the G2/M phase. In addition, a three-fold increase in the cell population in subG0 fraction after low concentration treatment with cucurbitacin B was seen in SKBR-3 compared to untreated cells (10.3% *vs*. 3.4%), implying apoptotic induction in these cells.

Over the last few years, telomerase has increasingly been seen as a viable target for cancer therapy, and as such there has been an increase in investigations seeking telomerase inhibitors as well as studies investigating the mode of action of these inhibitors. Inhibition of telomerase leads to progressive telomeric shortening during successive cell cycles and subsequently results in growth arrest and cell death [20].

Several reports have shown that telomerase activity can be inhibited by certain medicinal plants and extracts thereof. Medicinal plants are those in which some part of the plant can be used for a therapeutic purpose, and many of the drugs in use today are derived from medicinal plants [21]. Moreover, many studies seek to find new drug compounds from plants used in traditional medicine [22].

In 2005, Choi and colleagues reported that costunolide, a natural sesquiterpene compound derived from the bark of *Magnolia sieboldii* (a member of the Magnoliaceae family, which are commonly used in oriental traditional medicine [23]), inhibited the growth of a human breast cancer cell line by reducing telomerase activity [24]. Similarly, Ramachandran and colleagues reported that curcumin, derived from the rhizomes of turmeric (*Curcuma longa L.*)—a plant with a long history of natural medicinal usage—can inhibit activity of telomerase by down-regulating *hTERT* expression in MCF-7 breast cancer cells [25].

More recently, Mittal *et al.* reported the down-regulation of telomerase by (-)-epigallocatechin-3-gallate (EGCG), a constituent of green tea, in MCF-7 cells, leading to suppressed viability of the cell and the induction of apoptosis [26]. Inhibition of telomerase is therefore an attractive approach for breast cancer therapeutic goal, and medicinal plants seem to offer a wealth of potential candidate compounds.

In this work, we report the antiproliferative effect of a cucurbitacin extract derived from *Trichosanthes cucumerina L.* This plant is widely cultivated in Asia and the Indian sub-continent, where it has a long history of medicinal use for a variety of disorders, including malignant growths [27,28]. The extract, consisting of cucurbitacin B and dihydrocucurbitacin B, was shown to have significant antiproliferative effect on the human breast cancer cell lines tested (T47D, MCF-7, SKBR-3) as well as HBL-100 cells. Cell cycle blockages at G2/M phase as well as a certain degree of apoptosis were seen in these cell lines, especially in ER negative breast cancer SKBR-3 cells. Interestingly, the ER negative SKBR-3 cells were the most sensitive to cucurbitacin B when compared with the non-malignant HBL-100 cells and the other two breast cancer cells MCF-7 and T47D. As such, the different sensitivities to the cucurbitacin B compound seem to be directly related to estrogen receptor status, a finding that could be important for application of cucurbitacins to clinical practice.

The cucurbitacin B reported here affected telomerase activity as well as the expression of genes associated with regulation of this enzyme in all cell types studied. Expression of the *hTERT* gene is directly correlated with telomerase activity as the hTERT protein is the catalytic rate-limiting determinant subunit of telomerase [29]. *hTERT* is a downstream target of c-Myc factor which binds to E-box (CACGTG) and promotes expression of the *hTERT* gene [30]. Hence, c-Myc upregulates telomerase by enhancing *hTERT* expression [31,32]. In the estrogen positive cell lines T47D and MCF-7, however, the real time PCR showed some discordance between the reduction of expression of hTERT and c-Myc. This observation suggests that cucurbitacin B might exert its antiproliferative effects in ER positive and ER negative breast cancer cells via different biological pathways. Support for this comes from studies in human breast cancer cells that have shown that estradiol primarily regulates c-Myc at the level of transcription in ER positive cells, but at a post-transcriptional level in ER negative cells [33]. In ER positive cells it is known that the estrogen receptor activates a number of cellular signal transduction pathways, including the ERK/MAPK pathway [34] and activation of this pathway leads to the translocation of activated MAP kinase to the nucleus where it regulates the expression of a number of transcription factors, including pertinently, c-Myc [35]. Hence, in the ER positive cells, it is possible that cucurbitacin B directly modulates either the estrogen receptor or subsequent signaling pathway as is suggested by studies that shown the down regulation of the JAK/STAT pathway by cucurbitacin in pancreatic cells [36]. In ER negative cells where *c-Myc* is primarily controlled at the level of RNA stability [33], it is possible that cucurbitacin B reduces the half life of the RNA, leading to the loss of activation of *hTERT*. In both cases, cucurbitacin B could be acting through c-Myc, directly or indirectly, leading to the reduction of telomerase activity and subsequent loss of cellular proliferation, as confirmed by the finding of cell cycle G2/M blockage after cucurbitacin B treatment.

## 3. Experimental Section

### 3.1. Plant Material and Cucurbitacin Purification

Isolation of cucurbitacins from the fruit of *T. cucurmerina L.* was undertaken as described previously [19]. In this study three extracts were used; a crude spray dried extract of the fruit juice of *T. cucurmerina L*, a cucurbitacin extract containing cucurbititacin B and dihydrocucurbitacin B in an 8:1 molar ratio and purified cucurbititacin B [19]. Compounds and compound mixtures were dissolved in 10% dimethylsulfoxide (DMSO) and diluted with DMEM/F12 medium (DMEM/F12, Gibco, Grand Island, NY, USA) to the desired concentrations prior to use.

### 3.2. Cell Lines and Culture

Human breast cancer cell lines T47D, SKBR-3, MCF-7 and normal mammary epithelium cell line HBL-100 were purchased from the American Type Culture Collection (ATCC, Manassas, VA, USA) and were cultured in Dulbecco’s Modified Eagle’s Medium (DMEM/F12, Gibco, Grand Island, NY, USA) supplemented with 10% Fetal Bovine Serum (FBS, Gibco, Grand Island, NY, USA), 1% of penicillin streptomycin (Gibco, Grand Island, NY, USA) at 37 °C in a 5% CO_2_ incubator.

### 3.3. Cell Viability

Cell viability was assessed by the 3-(4,5-dimethylthiazol-2-yl)-2,5-diphenyltetrazolium bromide (MTT) assay, which is based on the conversion of MTT to MTT-formazan by mitochondrial enzymes as described elsewhere [37]. Cells (1 × 10^4^ cells/well) were seeded into a 96-well plate and allowed to attach to the well overnight. Compounds were added to a specified final concentration (1, 10, 20, 40, 60, 80 or 100 μg/mL) and cells incubated for a further 48 h. After incubation, 10 μL of 5 mg/mL 3-(4,5-dimethylthiazol-2-yl)-2,5-diphenyl tetrazolium bromide (MTT, Millipore, Billerica, MA, USA) was added to the cells for 4 h at 37 °C, followed by the addition of 100 μL isopropanol in 0.04 N HCl as the solubilizing agent. The absorbance at 570 nm was read using a microplate reader (Beckman coulter, Mississauga, ON, Canada) and the proportion of cell survival was calculated by dividing the average absorbance of the treated cells by the average of untreated cells. All experiments were performed in triplicate.

### 3.4. Telomerase Assay

Telomerase activity was measured by the PCR-based Telomeric Repeat Amplification Protocol (TRAP) assay [38,39], using the TRAPEZE ELISA Telomerase Detection Kit (Millipore, Billerica, MA, USA). The assay was performed following the manufacturer’s instructions. Briefly, 5 × 10^5^ cells per well were seeded into 6-well plates and incubated with cucurbitacin isolates at the specified concentration for 48 h. The cell pellets were then cooled on ice, lysed with CHAPS lysis buffer for 30 min and centrifuged at 12,000 × g at 4 °C for 20 min. The TRAP PCR reaction mixture contained 1 μg of protein from each cell extraction, 1 μL of biotinylated TS primer, 1 μL of RP primer, 1 μL of internal control (K1 primer and TSK1 template), 2.5 mM dNTPs and 2 units Taq DNA polymerase. The PCR was performed for 33 cycles of 94 °C for 30 sec, 55 °C for 30 s, 72 °C for 30 s and followed by a final extension at 72 °C for 10 min. The PCR products were separated by electrophoresis through 10% non-denaturing polyacrylamide gels to determine the degree of telomeric repeats and quantitated by ELISA to quantitatively determine telomerase activity.

For ELISA, 5 μL of the PCR product was mixed with 100 μL of the blocking buffer into a well of a streptavidin-coaded microtiter plate and the samples were incubated at 37 °C for 1 h. The blocking solution was completely removed and the well was rinsed 5 times with 250 μL of washing buffer. Afterthen, 100 μL of a horseradish peroxidase conjugated anti-dinitrophenyl (DNP) antibody was added to each well. The plate was incubated at room temperature for 30 min in a dark chamber. Antibody solution was removed and the wells were rinsed 5 times with 250 μL of washing buffer before the addition of 100 μL Tetramethylbenzidine (TMB). Samples were incubated for 10 min at room temperature before the addition of 100 μL of stop solution. Using a microplate reader, the amount of TRAP product was determined by measuring the absorbance ratio at 450 and 690 nm (reference wavelength) within 30 min of stopping the reaction.

### 3.5. RT-PCR

*Cells* (5 × 10^5^ cells/well) were seeded into 6-well plates and treated with cucurbitacin at various concentrations for 48 h. Total RNA was isolated from cell pellets using the RNeasy Mini Kit (QIAGEN, Hilden, Germany) in accordance with the manufacturer’s protocol. Following reverse transcription, cDNA was subsequently amplified by PCR with the primers shown in Table 2. The PCR conditions were as follows: for hTERT and β-actin, 35 cycles of denaturation at 95 °C for 30 s, annealing at 59.2 °C for 30 s and extension at 72 °C for 30 s. For c-Myc and β-actin, 30 cycles of denaturation at 95 ºC for 30 sec, annealing at 59.8 °C for 30 s and extension at 72 °C for 30 s. The PCR products were separated by electrophoresis through 0.8% agarose gels containing ethidium bromide and the PCR products were viewed under UV illumination. The PCR product bands were analyzed using GeneSnap software (SYNGENE, Frederic, MD, USA) and relative intensities were calculated after normalization against β-actin.

### 3.6. Realtime RT-PCR

Cells (5 × 10^5^ cells/well) were seeded into 6-well plates and treated with cucurbitacins at various concentrations for 48 h. Total RNA was isolated from cell pellets using the RNeasy Mini Kit (QIAGEN, Hilden, Germany) in accordance with the manufacturer’s protocol. Following reverse transcription, cDNA was subsequently amplified by realtime PCR, using SYBR-green in conjunction with the primers shown in Table 2. The PCR was analyzed using the iCycler iQ Multi-color Real-time PCR detection system (Bio-rad, Hercules, CA, USA). GAPDH was used as an internal control. All fold differences in the expression are expressed relative to GAPDH expression in the corresponding samples.

### 3.7. DNA Content/Cell Cycle Analysis

Cells were treated with cucurbitacins at various concentrations for 24 h and harvested. The cells were washed in 1 mL cold DPBS and fixed with 1 ml 75% ice-cold ethanol. After fixation, the cells were stained with 0.5 mL PI/RNase staining solution for 15 min in the dark. The stained cells were subjected to DNA content/cell cycle analysis using a FACSCaliber Flow cytometer system.

### 3.8. Statistical Analysis

Statistical analysis was performed using one-way ANOVA to compare between control and treated cells. P values < 0.05 were considered statistically significant.

## 4. Conclusions

Cucurbitacin B from *Trichosanthes cucumerina L* exerts a growth inhibitory effect in breast cancer cells. The most growth inhibition was seen in the ER negative breast cancer cell line SKBR-3. The mechanism of cucurbitacin B induced anticancer effects was addressed. In SKBR-3, cucurbitacin B blocks the cell cycle at G2/M even at low concentrations. Apoptotic induction was also suggested in this cell line according to the increased subG0 fraction after the treatment. Cucurbitacin B inhibits telomerase activity in the three breast cancer cell lines and an obvious inhibitory effect was seen in SKBR-3 cell rather than ER-positive MCF-7 and T47D cells. Expressions of hTERT and c-Myc were also inhibited by cucurbitacin B in these cells, and reduction of c-Myc protein was clearly observed in SKBR-3. In summary, anticancer bioactivities of cucurbitacin B are revealed by inhibiting telomerase via down regulation of both hTERT and c-Myc expression in breast cancer cells.

## Figures and Tables

**Table 1.** The half maximal inhibitory concentration (IC_50_) of three cucurbitacins extracted from *T. cucumerina L.* (Spray dried, cucurbitacin B major compound and pure cucurbitacin B) in breast cancer cell lines.

**Cells**	**IC_50_ (μg/mL) (Spray Dried)**	**IC_50_ (μg/mL) (CuB Compound)**	**IC_50_ (μg/mL) (Pure CuB)**	**Cell’s Estrogen Receptor (ER) status**
SKBR3-3	<10	4.6	3.29	Neg
HBL-100	73.7	55.4	32.77	Neg
MCF-7	>100	88.75	63.07	Pos
T47D	>100	>100	88.4	Pos

**Table 2.** The primer sequences for RT-PCR and Realtime PCR.

**Genes**	**Primers**		**Fragment size (bp)**

	**Forward**	**Reverse**	
**RT-PCR**			
hTERT	5′-CGGAAGAGTGTCTGGAGCAA-3′	5′-GGATGAAGCGGAGTCTGGA-3′	145
c-Myc	5′-AAGTCCTGCGCCTCGCAA-3′	5′-GCTGTGGCCTCCAGCAGA-3′	249
β-actin	5′-GCTCGTCGTCGACAACGGCT-3′	5′-CAAACATGATCTGGGTCATCTTCTC-3′	353
**Real-time PCR**			
hTERT	5′-TCCACTCCCCACATAGGAATAGTC-3′	5′-TCCTTCTCAGGGTCTCCACCT-3′	110
c-Myc	5′-ACCACCAGCAGCGACTCTGA-3′	5′-TCCAGCAGAAGGTGATCCAGACT-3′	117
GAPDH	5′-GAAGGTGAAGGTCGGAGTC-3′	5′GAAGATGGTGATGGATTTC-3′	225
